# Conjugation of Polymer-Coated Gold Nanoparticles with Antibodies—Synthesis and Characterization

**DOI:** 10.3390/nano5031297

**Published:** 2015-08-07

**Authors:** Gamze Tan, Karsten Kantner, Qian Zhang, Mahmoud G. Soliman, Pablo del Pino, Wolfgang J. Parak, Mehmet A. Onur, Daniel Valdeperez, Joanna Rejman, Beatriz Pelaz

**Affiliations:** 1 Faculty of Science and Letters, Department of Biology, Aksaray University, Aksaray 68100, Turkey; E-Mail: gamzetan2003@yahoo.com; 2 Philipp University of Marburg, Marburg 35001, Germany; E-Mails: karsten.kantner@physik.uni-marburg.de (K.K.); qian.zhang@physik.uni-marburg.de (Q.Z.); mahmoud.gamalsoliman@physik.uni-marburg.de (M.G.S.); wolfgang.parak@physik.uni-marburg.de (W.J.P.); daniel.valdepereztoledo@physik.uni-marburg.de (D.V.); 3 Centro de Investigación Cooperativa Biomagune, San Sebastián 20001, Spain; E-Mail: pdelpino@cicbiomagune.es; 4 Faculty of Science, Department of Biology, Hacettepe University, Ankara 06800, Turkey; E-Mail: mali@hacettepe.edu.tr

**Keywords:** gold nanoparticles, bioconjugation, nanoparticle characterization, toxicity, nanoparticle-cell interaction, cellular uptake, VEGF

## Abstract

The synthesis of polymer-coated gold nanoparticles with high colloidal stability is described, together with appropriate characterization techniques concerning the colloidal properties of the nanoparticles. Antibodies against vascular endothelial growth factor (VEGF) are conjugated to the surface of the nanoparticles. Antibody attachment is probed by different techniques, giving a guideline about the characterization of such conjugates. The effect of the nanoparticles on human adenocarcinoma alveolar basal epithelial cells (A549) and human umbilical vein endothelial cells (HUVECs) is probed in terms of internalization and viability assays.

## 1. Introduction

The synthesis in colloidal nanoparticles (NPs) is well advanced [1,2,3,4,5,6]. Nowadays, a high control concerning material composition, size, shape, *etc.*, is possible [7]. There are also many strategies available to provide water-solubility of these NPs with high colloidal stability [8]. Some correlation of the (nonspecific) interaction of such NPs with cells with their physicochemical properties is possible and some general tendencies are well accepted in literature [9,10]. However, in order to warrant for specific interaction of NPs with cells, their surface has to be modified with ligands targeting cellular receptors. The purpose to bind proteins to the surface of NPs is to provide them a special ligand coat that they interact specifically with cells, *etc.* While there are many reports in literature about the conjugation of NP surfaces with specific ligands, characterization of these NPs is not always sufficient. Bioconjugation in particular may result in unwanted agglomeration, due to crosslinking of NPs. Thus, characterization of the colloidal properties of such conjugates is of high importance. In addition, the ligand density may significantly vary, depending on the used conjugation protocol. In principle, solutions to these hurdles exist, and NPs with a controlled ligand density and controlled ligand orientation can be synthesized [11,12,13]. However, these synthesis strategies require typically sophisticated protocols, and thus most commonly in literature more simple and less controlled strategies are employed. In the present work, it will be shown that also by simple conjugation strategies, together with appropriate characterization techniques, NP-antibody conjugates can be generated. As, in particular, characterization is crucial in the following, all experimental steps will be presented in the form of a general protocol.

## 2. Materials and Discussions

### 2.1. Synthesis of Gold Nanoparticles

Au NPs are standard model systems, which are extensively used in literature to study the interaction of NPs with cells. This is in particular due to the fact that Au is an intrinsically nontoxic material. In the following, a protocol for the synthesis of hydrophilic Au NPs is described according to standard protocols from literature [14,15,16,17].

For the synthesis of Au NPs of *d_c_* = 20 nm core diameter, hydrogen tetrachloroaurate (III) hydrate (Alfa Aesar #12325, Ward Hill, MA, USA) and sodium citrate dehydrate 99% (Sigma Aldrich #W302600, St. Louis, MS, USA) were used as chemicals. All chemicals were used without further purification. Ultrapure water with a resistance greater than 18.2 mΩ·cm^−1^ was used for all experiments. All glassware was cleaned in aqua regia and rinsed with ultrapure water. For the synthesis, a solution containing 150 mL (2.2 mM) trisodium citrate dihydrate (Na_3_C_6_H_5_O_7_·2H_2_O) was heated in a 250 mL flask to 100 °C with stirring under reflux. Using a syringe, 1 mL of 25 mM HAuCl_4_·3H_2_O was injected into the flask and stirred at 100 °C. Upon formation of Au NPs, the solution turned deep red. The temperature was then reduced to 90 °C, and the solution was stirred continuously for another 30 min. For further NP growth, then 1 mL sodium citrate (60 mM) and 1 mL of HAuCl_4_ solution (25 mM) were sequentially injected with a time delay of two minutes between the two injections [17]. After 30 min, the reaction was cooled down to room temperature using an ice bath.

While this protocol virtually always will lead to the formation of Au NPs (as visible by the red color of the solution) the quality of the NPs can vary significantly. Concerning colloidal solutions, the two most important quality indicators are dispersion (*i.e.*, the NPs are individually dispersed and do not agglomerate) and size distribution of the NPs (*i.e.*, the diameter of all NPs should be as similar as possible). Even by using the same synthesis protocol over and over, the quality of the resulting NPs may vary for each batch, which warrants a mandatory quality control.

**Figure 1:**
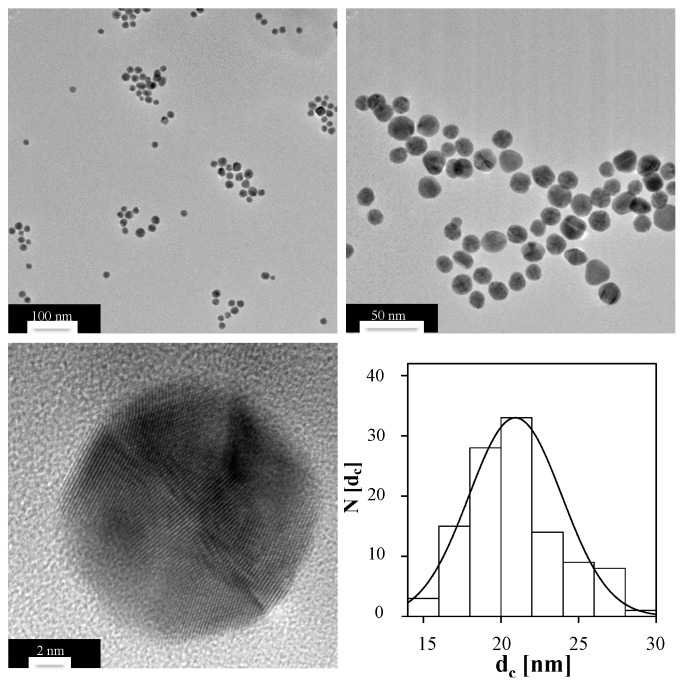
TEM images of the Au NPs at different magnifications (scale bars corresponding to 100 nm, 50 nm and 2 nm), and the corresponding histogram N (*d_c_*) of the core diameter *d_c_*.

The size distribution of inorganic NPs, *i.e.*, NPs with a core composed out of an inorganic material such as gold, can be determined with transmission electron microscopy (TEM). Note that organic molecules often do not provide sufficient contrast for being visualized with TEM. For TEM analysis, a diluted drop of Au NPs was dried on a copper grid, and NPs were imaged with TEM. From such images (*cf.* Figure 1), a histogram about the distribution of the core diameter, *i.e.*, the diameter *d_c_* of the inorganic NP core can be obtained. In the present case, the core diameter was determined by analysis of more than 300 NPs, using the free software Image J. From the histogram, the mean diameter of the Au cores was determined to be *d_c_* = 20.9 ± 4.3 nm, *cf.* Figure 1.

**Figure 2:**
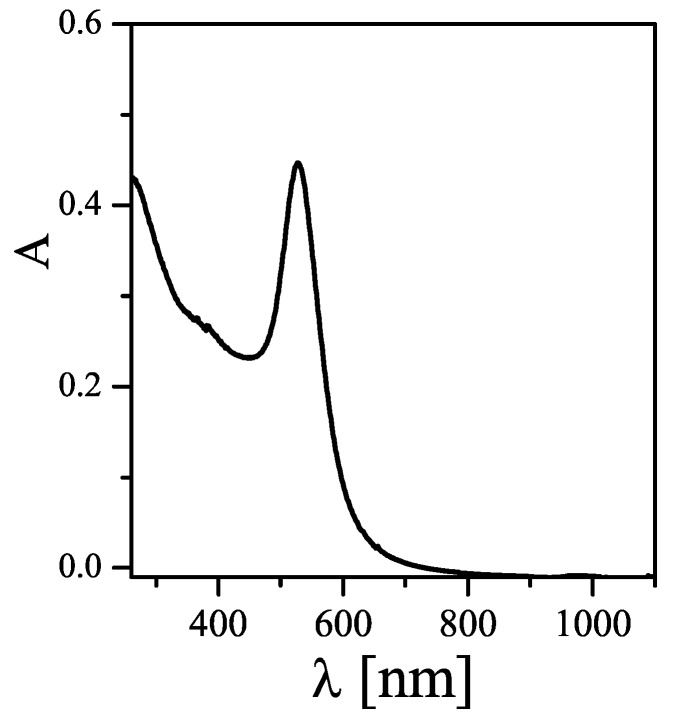
UV/Vis absorption spectrum A (λ) of Au NPs dispersed in water, directly recorded after their synthesis as described in this chapter.

The state of dispersion cannot be unequivocally deduced from TEM images, as those are recorded on NPs in dried state. In other words, even well dispersed NPs can form clusters on TEM grids. While the most common method to probe for NP dispersion is measuring the hydrodynamic diameter directly in solution (for example by dynamic light scattering (DLS), as will be described later in more detail), in the case of Au NPs simple analysis can be done by recording UV/Vis absorption spectra. As shown in the absorption spectrum in Figure 2, Au NPs exhibit a peak due to surface plasmon resonance [18]. In case NPs are not well dispersed and start to form agglomerates, this peak is shifted to higher wavelengths and the solution turns from red to blue-black. Agglomeration also leads to scattering at high wavelengths >800 nm. In case of poor size distribution, the plasmon peak broadens.

Besides giving an indication about the quality of the NP synthesis, UV/Vis absorption spectra are also helpful for the determination of the concentration of the Au NPs. According to the Lambert-Beer Law, the absorption *A* of a solution of NPs (with a length of the light path *L*) is proportional to the NP concentration *c_NP_*:

(1) $$A={ɛ}_{\text{NP}}\text{·}L\text{·}c_{NP}$$

The proportionality factor is the molar extinction coefficient, which is well determined in the case of Au NPs with different sized. In the present case of NPs with a core diameter of *d_c_* ≈ 20 nm the extinction coefficient at 450 nm is given as ɛ_NP_ (450nm) = 5.41× 10^8^·M^−1^·cm^−1^ [19]. For the present case, 20 μL of Au NP solution directly taken after their synthesis, after dilution 500 µL with water, lead to an absorbance of *A* = 0.23 at 450 nm (*L* = 1 cm). That means that the Au NP concentration was around *c_NP_* ≈ 11.1 nM.

As citric acid capped Au NPs as prepared above are not highly colloidally stable in cell culture media (due to screening of their surface charge by adsorption of counter ions), the NPs were further stabilized by modification with polyethyleneglycol (PEG) [20]. In this work, the as-prepared Au NPs were modified with a heterofunctional PEG chain with a thiol group at one, and a carboxylic group at the other end (molecular mass *M*_w_ = 3 kDa, Rapp polymer #133000-4-32, Tuebingen, Germany). 10^5^ PEG molecules were added *per* each NP, and the pH was risen to 12 with NaOH (1 M). Alkaline conditions facilitate deprotonation of the thiol terminal, which, in this way, attaches faster to the Au surface [21]. Afterwards, the PEGylated NPs were cleaned by centrifugation in order to remove unbound PEG (three times using 14,000 rpm for 30 min, supernatant containing free PEG is discarded and replaced by fresh buffer).

### 2.2. Fluorescence Labelling of Proteins

**Figure 3:**
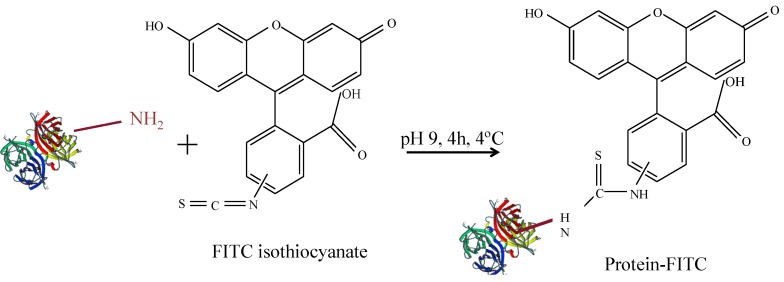
Scheme for FITC-labelling of proteins.

Protein concentrations are often determined by absorption measurements, for example by the Bradford assays, as described later. However, as NPs heavily absorb in the same range of wavelengths absorption measurements are not well suited for determining protein concentrations in NP-protein conjugates. In contrast, in order to quantify protein conjugation to NPs, it is useful to label proteins with a fluorophore. In this way, protein concentration can be determined by measuring fluorescence emission intensities. In the following, a protocol for conjugation of proteins with fluorescein isothiocyanate (FITC) is given. FITC can be directly linked to the proteins as depicted in Figure 3 [22].

**Figure 4:**
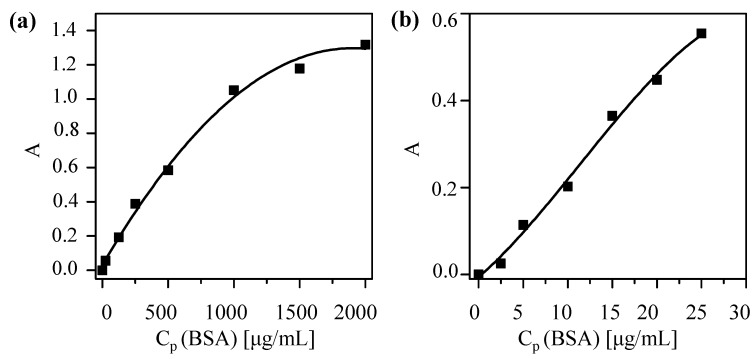
Coomassie assay calibration curves to determine the protein concentration *C_P_* of solutions with (**a**) high concentration and (**b**) low concentrations by measuring the offset-corrected absorption *A* at 595 nm. The fitting curves are (**a**) *A*(*C_P_*) = 0.029 + (0.001 mL/μg)·*C_P_* − (3 × 10^−7^ mL^2^/μg^2^)·*C_P_*^2^, and (**b**) *A*(*C_P_*) = −0.005 + (0.017 mL/μg)·*C_P_* – (6.789 × 10^−4^ mL^2^/μg^2^)·*C_P_*^2^ – (1.97 × 10^−5^ mL^3^/μg^3^)·*C_P_*^3^ and the coefficients of determination (*r*^2^) are equal to 0.994 and 0.989, respectively.

First, a calibration curve based on the Bradford assay [23] (Coomassie Blue, Thermo Scientific #23236, Hampton, NH, USA) to determine protein concentrations was obtained. Under the presence of proteins, a shift in the absorption spectrum of Coomassie Blue occurs and protein concentration is proportional (in a certain range) to the (offset-corrected) absorption A at 595 nm. The calibration curve was done following the fabricant specifications [24]. As protein standards, bovine serum albumin (BSA) was used (Thermo Scientific #23029). Two different calibration curves were recorded, one for high protein concentrations (working range of 100–1500 μg/mL protein concentrations *C_P_*) and a second one for low protein concentrations (working range 1–25 μg/mL protein concentrations *C_P_*). The standard solutions of different protein concentrations *C_P_* were prepared as indicated in the protocol, using 2-(*N*-Morpholino)ethanesulfonicacidhydrate (MES) pH 6.5 as buffer. Following the indication of the guide, in order to get the high concentration curve, 10 μL of NP solution sample were mixed with 300 μL of Coomassie reagent, previously equilibrated at room temperature. To obtain the low concentration curve instead of using 10 μL NP sample and 300 μLCoomassie reagent, 150 μL of sample and 150 μL of reagent were used. After mixing for 30 s and incubating for 10 min for each protein concentration, *C_P_*, the absorption of the protein—Coomassie Blue mix at 595 nm—was recorded with an UV/Vis absorption spectrometer (Agilent 8450 spectrometer, Palo Alto, CA, USA). Single-use plastic cuvettes were used to hold the samples. Samples were prepared by triplicate and measured individually. As an offset, the absorption of Coomassie Blue without protein was subtracted. The offset-corrected absorptions A are plotted *versus* the protein concentrations *C_P_* in Figure 4. A polynomial fit was applied to obtain the final calibration curves.

**Figure 5:**
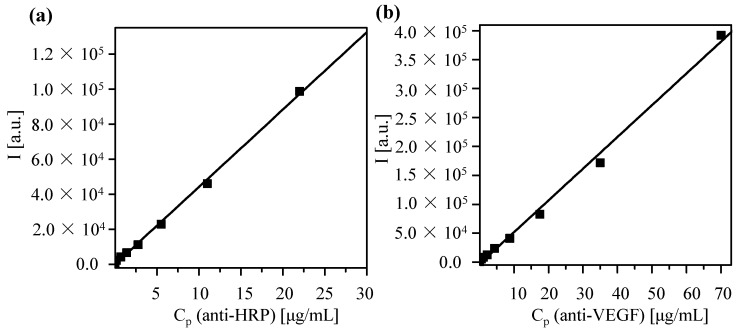
Calibration curve in which the fluorescence intensity *I* at 519 nm is determined for protein solutions of different concentration *C_P_*. Data are shown for antibodies against (**a**) horseradish peroxidase (HRP) and (**b**) vascular endothelial growth factor (VEGF). A linear fit leads to the following correlation between fluorescence intensity *I* and concentration *C_P_*: (**a**) *I*(C_P_) = *I*_0_ + (Δ*I*/ΔC_P_)·*C_P_* = −464.28 + (4447.1 mL/µg)·*C_P_*; (**b**) *I*(*C_P_*) = −3549.4 + (5498.3 mL/µg)·*C_P_*. The coefficients of determination (*r*^2^) are equal to 0.998 and 0.995 for HRP and VEGF, respectively.

FITC conjugation was performed using the following protocol. First, the concentration of proteins was determined with the Bradford method as described above. Then, a FITC stock solution was prepared in sodium borate buffer (SBB) at pH = 9, equaling 750 FITC molecules *per* protein. FITC was added to the proteins and the mixture was incubated for at least 4 h at 4 °C. For removal of unbound FITC, the sample was run through a PD 10 or a PD 25 column (depending on the solution volume, GE Healthcare #52-1308-00 and #28-9180-07, respectively, Little Chalfont, UK) and only the protein containing fraction was collected. After the column purification the protein solution becomes diluted, the protein concentration *C_P_* (of the now FITC-conjugated proteins) was determined again with the Bradford assay. A dilution series of the proteins was obtained and, for each protein concentration, the fluorescence intensity *I* at 519 nm (the emission wavelength of FITC) was determined. By plotting, the fluorescence intensity *versus* the protein concentration as calibration curve was obtained, *cf.* Figure 5.

### 2.3. Conjugation of NPs with Proteins

**Figure 6:**
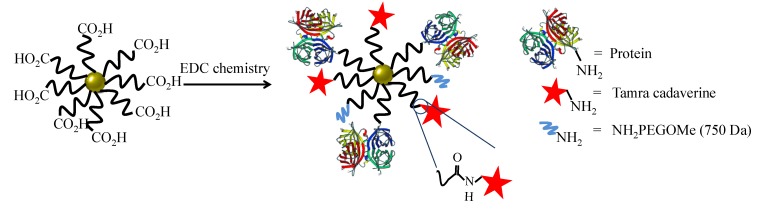
Scheme of the NP modification with antibody, dye and PEG (as passivating agent).

Here, an often used strategy based on *N*-(3-Dimethylaminopropyl)-*N*′-ethylcarbodiimide hydrochloride (EDC, Sigma Aldrich) was employed [22]. Note that while EDC chemistry is straightforward for the formation of peptide bonds between amine groups (here present on the protein ligands) and carboxyl groups (here present on the NP surface at the PEG terminal pointing towards solution), it may result in the formation of agglomerates, and thus characterization of the resulting conjugates is required. In addition, amine groups which belong to the functional part of the proteins can be deactivated upon linkage (reaction will occur statistically on the present amine groups of the proteins), and some proteins may lose their biological activity—in the present case, antibodies against HRP (anti-peroxidase, Sigma Aldrich) or against VEGF (anti-VEGF, R&D systems, AB-293-NA) where they are linked to the NPs. As described above, the antibodies were optionally tagged with FITC. In addition to the proteins, 5-(6)-carboxytetramethylrhodaminecadaverine (“TAMRA”, Anaspec #81507, Fremont, CA, USA) was also attached as additional fluorophore via its amine group to the NP surface. Third ligand short metoxy-PEG-amine (amine-PE; *M*_w_ = 750 kDa, Rap Polymer #12750-2, Tuebingen, Germany) was attached via its amine group to the NP surface, in order to preserve the activity of the antibodies [25] and to prevent nonspecific protein absorption [26,27]. In other words, three different ligands (proteins, TAMRA, PEG) were attached to the PEGylated NPs using EDC chemistry. The ratios were chosen that *per* 1 Au NP 7.5 × 10^6^ EDC molecules, 50 antibodies, 10^3^ TAMRA molecules, and 2.5 × 10^4^ amine-PEG molecules were added for reaction. The reaction scheme is presented in Figure 6.

For the reaction 19.23 μL of Au, NPs dispersed in water (corresponding to 4pmol) with concentration *c_NP_* = 208 nM were taken, and mixed with 923.5 μL of 4-morpholineethanesulfonic acid (MES, Sigma Aldrich #M8250, 50 mM, pH 6.5) and 57.3 µL of EDC stock solution (100 mg/mL). After 20 min, the sample with a total volume *V* = 1 mL containing the activated NPs was cleaned from unreacted EDC and the salts, using a pre-packed column PD-10 desalting column (GE healthcare #17-0851-01, Bucks, UK) equilibrated with MES (50 mM, pH 6.5). During this step, the NP volume was roughly diluted twice. In addition, using a high pH such as 8 was tried, with the motivation to take advantage of linking antibodies in an oriented manner [28], but the activation process was not working as well as at pH 6.5, and thus, throughout this work, pH 6.5 was used. The volume of the eluted NP solution was adjusted with MES buffer to 2 mL. Immediately after the NP cleaning, 30 μg of antibodies were added. After incubation for 15 min, 2 μg of TAMRA were added. Finally, after another 15 min of incubation, 75 μg of amine-PEG were added to block the remaining reactive carboxylic groups. The reaction mixture was incubated for another 1 h at room temperature and then incubated at 4 °C overnight. Unbound proteins, dye molecules, and PEG were removed by repetitive centrifugation (14,000 rpm 30 min), until no fluorescence was detected in the supernatant. This required at least five cleaning cycles (pelleting of NPs, discarding of supernatant, resuspending the NP pellet in fresh buffer). In the first washing step, 10 μL of sodium dodecylsulfate (SDS, 10%) was added to remove nonspecifically adsorbed dyes or proteins. Following this protocol, NPs conjugated with anti-HRP or anti-VEGF (with optional FITC label) were synthesized. As a control, the reaction was carried out without adding antibodies, but only TAMRA and PEG, leading to control NPs. In the following, the PEGylated Au NPs before bioconjugation will be referred to as Au-PEG NPs. The NPs after bioconjugation with anti-HRP, anti-VEGF, or without having antibody added will be referred to as Au-PEG-anti-HRP NPs, Au-PEG-anti-VEGF NPs, or Au-PEG-control NPs. In case the antibodies had been labelled with FITC, this is indicated as “*”: Au-PEG-anti-HRP* NPs, Au-PEG-anti-VEGF* NPs.

In the vicinity of the Au surface, organic fluorophores may be quenched. Distance dependent measurements have been demonstrated that quenching can occur well up to separation distances of the fluorophores from the Au surface of 10 nm [29]. In the present work, no direct contact of fluorophores with the Au surface is possible due to the layer of 3 kDa PEG. This layer will keep the fluorophores at ≈4 nm distance to the Au surface [30]. In the case of TAMRA, conjugation directly to the PEG terminal pointing towards solution quenching does not impose any problem, as no quantitative fluorescence measurements are performed. The TAMRA merely serves as a label for qualitative fluorescence imaging of NPs that have been internalized by cells and thus quenching does not interfere with experiments. In the case of the FITC-labelled proteins, partial quenching of their fluorescence upon binding to the surface of the PEGylated Au NPs cannot be excluded. However, the proteins will randomly orient on the NP surface. Only in the case that the FITC attached to the protein is oriented towards the NP surface, significant quenching is expected, as in the case FITC attached to the protein is oriented towards solution, away from the NP surface, the distance between FITC and the Au surface is further increased by the size of the protein. Together with the PEG spacer, which is always present, one clearly cannot exclude quenching, though it is not estimated to play a huge role. Due to quenching, there is less fluorescence signal from proteins attached to the NP surface as in comparison to the fluorescence of the free proteins, which have been used for obtaining the calibration curve. In this way, in the procedure described here, the number of proteins attached per NP is underestimated.

### 2.4. Determination of the Number of Antibodies Bound per NP

The number of antibodies per NP (*R_P/NP_*) can determined from separately measuring the protein concentration *c_P_* and the NP concentration *c_NP_* of NP-antibody conjugates:

(2) $$R_{P/NP}=c_{P}/c_{NP}$$

**Figure 7:**
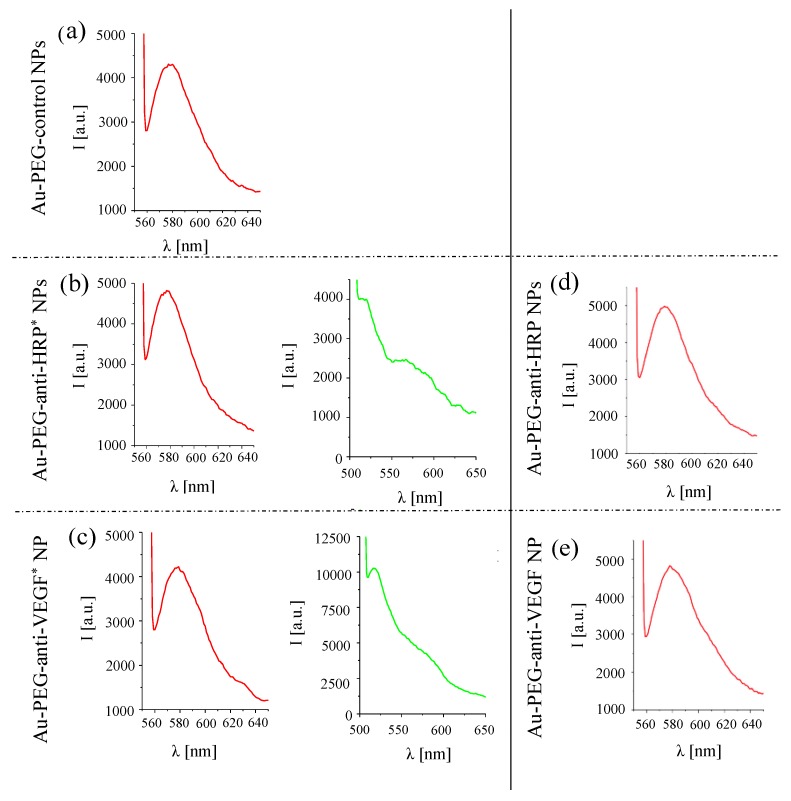
Left side: Fluorescence spectra recorded for (**a**) Au-PEG-control NPs, (**b**) Au-PEG-anti-HRP* NPs, and (**c**) Au-PEG-anti-VEGF* NPs at a NP concentration of *c_NP_* = 2 nM. Excitation was performed at 545 nm (TAMRA, drawn in red) or at 494 nm (FITC, drawn in green). Fluorescence spectra were recorded under the same conditions as the spectra recorded for the calibration curve Figure 5. Right side: Fluorescence spectra recorded for (**d**) Au-PEG-anti-HRP NPs, and (**e**) Au-PEG-anti-VEGF NPs for TAMRA excitation (545 nm) at a NP concentration of *c_NP_* = 2 nM.

The NP concentration can be obtained from the absorption spectra of the conjugates at the wavelengths of the surface plasmon peak, at which the antibodies barely absorb. The protein concentration is determined from fluorescence spectra (*cf.* Figure 7) and the calibration curve shown in Figure 4.

Upon excitation of FITC (at 494 nm), there is also some fluorescence of TAMRA, which however can be clearly distinguished from the FITC fluorescence (*cf.* the green curve in Figure 7). From the FITC fluorescence spectra (*cf.* the green curve in Figure 7), the emission *I* at 519 nm was determined. Based on the calibration curve given in Figure 5, the protein concentration *C_P_* can be determined as:

(3) $$C_{P}=(I\text{−}I_{0})/(\Delta I/\Delta C_{P})$$

using the fit parameters *I*_0_ and Δ*I*/Δ*C_P_* from the calibration curve given in Figure 5. The mass concentration *C_P_* of the proteins can be converted in molar concentrations *c_P_* by using the molecular mass *M*_W_ of the proteins: *c_P_* = *C_P_*/*M*_w_. The results as obtained for the Au-PEG-anti-HRP* and Au-PEG-anti-VEGF* NPs are given in Table 1, based on the data shown in Figure 5 and Figure 7. For each sample, two different dilutions were measured.

**Table 1:** Summary of the calculations for the amount of proteins per nanoparticles (NP).

Sample	Au-PEG-anti-HRP* NPs	Au-PEG-anti-HRP* NPs	Au-PEG-anti-VEGF* NPs	Au-PEG-anti-VEGF* NPs
*c_NP_* (nM)	2.2	1.5	2.0	0.5
*M*_W_ (g/mol)	150,000	150,000	150,000	150,000
Δ *I*/Δ*C_P_* (mL/μg) (*cf.* Figure 5)	4,447.1	4,447.1	5,498.3	5,498.3
*I*_0_ = *I* (*C_P_*= 0) (a.u.) (*cf.* Figure 5)	−464.28	−464.28	−3,549.4	−3,549.4
*I* (a.u.) (*cf.* Figure 7)	3,999	2,420	10,212	2,600
*C_P_* (μg/mL)	1.0	0.65	2.5	1.12
*c_P_* (nM)	6.67	4.33	16.67	7.47
*R_P_*/*_NP_*	2.7	2.4	6.2	6.3

### 2.5. Physicochemical Characterization of the NP-Antibody Conjugates

**Figure 8:**
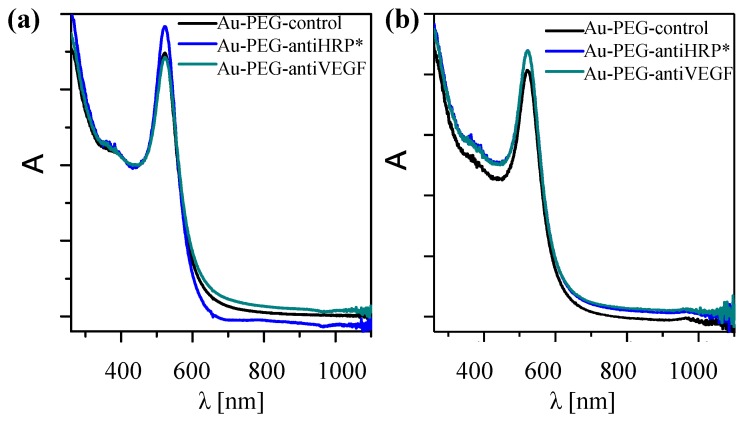
Normalized UV/Vis spectra of the NP-antibody conjugates. (**a**) Au-PEG-control, Au-PEG-anti-HRP*, and Au-PEG-anti-VEGF* NPs; (**b**) Au-PEG-control, Au-PEG-anti-HRP, and Au-PEG-anti-VEGF NPs. Spectra were recorded in a spectrometer Agilent 8453.

As already mentioned, bioconjugation may change the colloidal properties of NPs. Thus, characterization should be also performed with the resulting samples. The UV/Vis absorption spectra shown in Figure 8 indicate that upon bioconjugation, no significant agglomeration occurred, as scattering for wavelengths >800 nm can be neglected.

**Figure 9:**
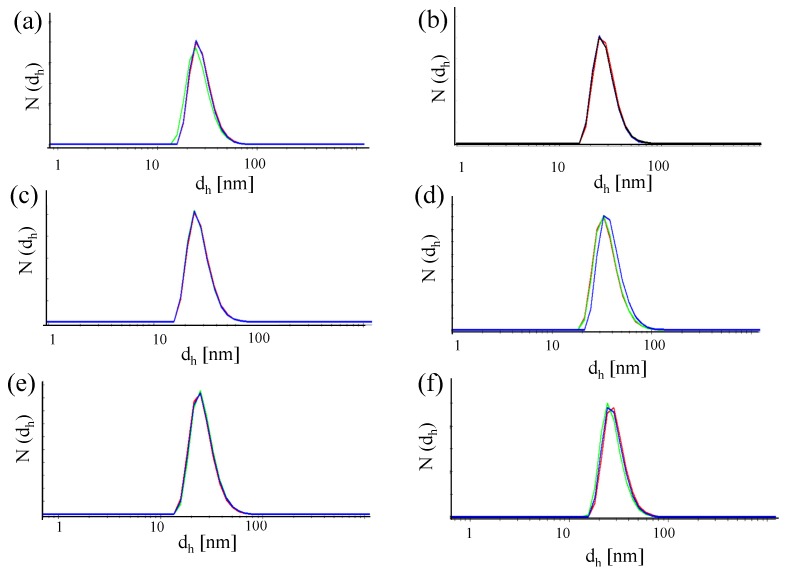
Number distribution N (*d_h_*) of the hydrodynamic diameter recorded or different NPs. (**a**) Au-PEG NPs, (**b**) Au-PEG-control NPs, (**c**) Au-PEG-anti-HRP* NPs, (**d**) Au-PEG-anti-VEGF* NPs, (**e**) Au-PEG-anti-HRP NPs, and (**f**) Au-PEG-anti-VEGF NPs. The concentration of the NP solutions were *c_NP_* ≈ 5 nM, and the measurements were performed in milliQ water. Each sample was measured at least three times and the mean value of the hydrodynamic diameter was determined.

While UV/Vis absorption spectra can be a first indication about the presence of bigger agglomerates, it is hard to determine the existence of smaller agglomerates from these data. For this purpose, measurements of the hydrodynamic diameter *d_h_* of the NPs are best suited. One common technique in this direction is dynamic light scattering (DLS; Malvern Zetasizer set-up). However, in the case of small NPs, proteins have similar size to the NPs and thus measurements in cell culture media containing serum are complicated [31]. In Figure 9, DLS measurement for the NP-antibody conjugates are displayed. The hydrodynamic diameters *d_h_* as determined from these data (*cf.* Table 2) demonstrate that any larger agglomerates can be excluded. However, in general, no significant increase in size of the NPs upon antibody attachment could be observed, though the FITC fluorescence clearly proves the presence of the antibodies.

**Figure 10:**
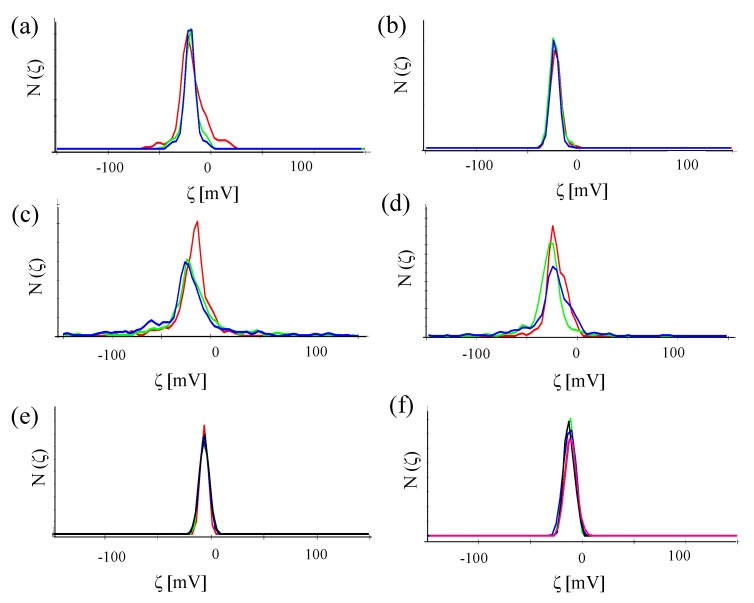
Number distribution N (ζ) of the zeta-potential of (**a**) Au-PEG NPs, (**b**) Au-PEG-control NPs, (**c**) Au-PEG-anti-HRP* NPs, (**d**) Au-PEG-anti-VEGF* NPs, (**e**) Au-PEG-anti-HRP NPs, and (**f**) Au-PEG-anti-VEGF NPs. The concentration of the NP solutions were *c_NP_* ≈ 5 nM, and the measurements were performed in milliQ water. Each sample was measured at least three times and the mean value of the zeta-potential was determined.

In the same Malvern Zetasizer set-up, the zeta-potential ζ was also determined based on laser Doppler anemometry, *cf.* Figure 10. The data shown in Table 2 show that despite attachment of antibodies, the NPs retained their negative zeta-potential. In the case of conjugation with antibodies without FITC, the NP-antibody conjugates have a less negative zeta potential than the NPs without attached antibodies.

As proteins can also nonspecifically adsorb to the surface of NPs, the conjugation reactions were repeated but without addition of EDC. In this way, all attached proteins are not covalently attached (as due to the lack of EDC, no amide bonds can be formed), but are nonspecifically attached to the NPs. These samples are termed Au-PEG/control, Au-PEG/anti-HRP, and Au-PEG/anti-VEGF NPs. Hydrodynamic diameters and zeta-potentials as determined with these NPs are enlisted in Table 3. There is less reduction of negative zeta potential upon presence of the antibodies. Thus, less antibodies are present per NP and, in this way, attachment of antibodies under the presence of EDC should be mainly of covalent nature.

**Table 2:** Summary of mean hydrodynamic diameters and zeta-potentials determined in water.

Sample	Au-PEG NPs	Au-PEG-Control NPs	Au-PEG-anti-HRP* NPs	Au-PEG-anti-VEGF* NPs	Au-PEG-anti-HRP NPs	Au-PEG-anti-VEGF NPs
*d_h_* (nm)	27.4 ± 0.4	27.8 ± 0.8	28.0 ± 0.2	38.0 ± 1.9	27.0 ± 0.6	28.9 ± 0.9
ζ (mV)	−32.8 ± 0.6	−20.0 ± 0.9	−18.4 ± 1.6	−24.1± 3.8	−6.3 ± 0.2	−11.8 ± 0.7

**Table 3:** Summary of mean hydrodynamic diameters and zeta-potentials determined in water.

Sample	Au-PEG NPs	Au PEG-Control NPs	Au-PEG-anti-HRP NPs	Au-PEG-anti-VEGF NPs
*d_h_* (nm)	27.4 ± 0.4	27.4 ± 1.6	26.1 ± 2.1	29.3 ± 1.7
ζ (mV)	−32.8 ± 0. 6	−30.1 ± 1.2	−24.3± 0.9	−31.4± 1.3

### 2.6. NP Interaction with Cells

NPs can be internalized by cells via endocytosis, as they may trigger toxic effects. In the following, this is investigated for two different cell lines, human adenocarcinoma alveolar basal epithelial cells (A549) and human umbilical vein endothelial cells (HUVECs). A549 cells, purchased from ATCC, were cultured in Dulbecco’s Modified Eagle’s Medium (DMEM, Sigma Aldrich) supplemented with 10% fetal bovine serum, 2 mM l-glutamine (Sigma Aldrich), and 100 U/mL penicillin/streptomycin (Sigma Aldrich). HUVECs, purchased fromPromoCell, were cultured in Endothelial Cell Basal Medium (ECBM, PromoCell, Heidelberg, Germany) supplemented with 2% fetal calf serum (PromoCell), 0.4% Endothelial Cell Growth Supplement (PromoCell), Epidermal Growth Factor (0.1 ng/mL, PromoCell), Basic Fibroblast Growth Factor (1 ng/mL, PromoCell), heparin (90 μg/mL, PromoCell) and hydrocortisone (1 μg/mL, PromoCell). The cells were grown at 37 °C in a humidified atmosphere containing 5% CO_2._

**Figure 11:**
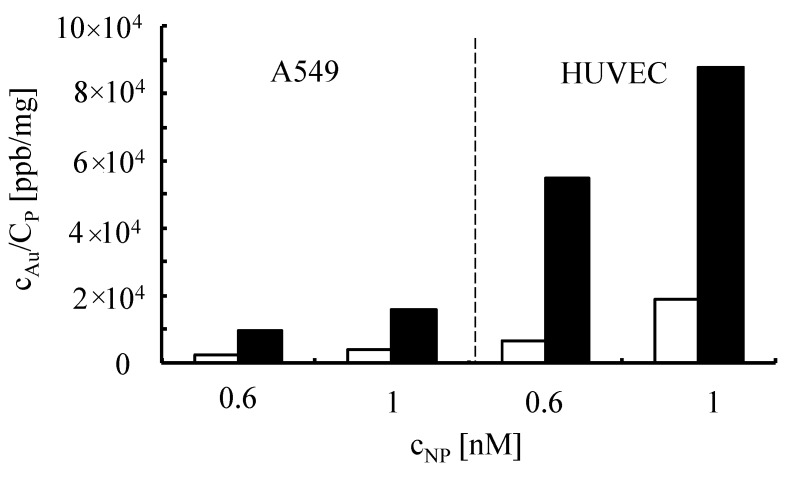
Internalization of Au-PEG NPs by A549 cells and HUVECs. A549 cells and HUVECs were incubated with Au-PEG NPs of *c_NP_* = 0.6 nM and *c_NP_* = 1 nM concentration cells in medium with (white bars) or without (black bars) serum. Twenty-four hours after adding the particles, the gold concentration *c_Au_* and the protein concentration *C_P_* was determined.

For uptake experiments, cells were incubated with NPs and after 24 h the amount of incorporated NPs was determined. A549 cells and HUVECs were incubated with Au-PEG NPs of different concentration within medium with or without serum. After 4 h of incubation, the cells were intensively washed and further cultured. Since serum components are known to alter physicochemical characteristics of NPs, we studied their internalization in the presence and absence of serum. Twenty-four hours after adding the NPs, the cells were lysed and the samples were analyzed for their gold content with inductively coupled plasma mass spectrometry (ICP-MS). The protein content of each sample was determined by the Bradford assay (Bio-Rad, Hercules, CA, USA). The results are presented in Figure 11 as ppb of gold per mg of protein.

**Figure 12:**
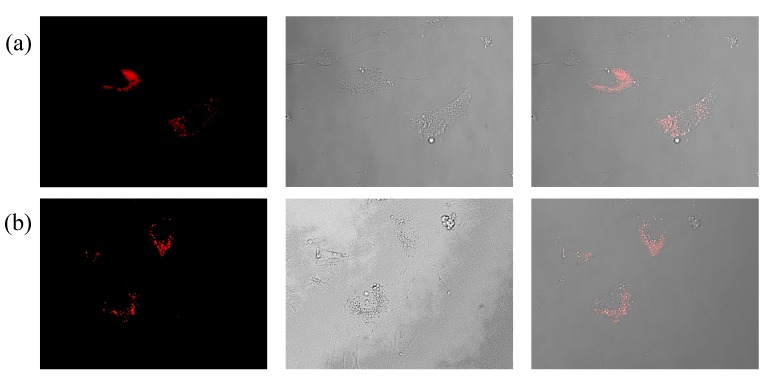
Internalization of functionalized Au NPs by human umbilical vein endothelial cells. HUVECs were exposed to TAMRA-labeled Au NPs functionalized with (**a**) anti-VEGF (Au-PEG-antiVEGF NPs), and (**b**) anti-HRP antibodies (Au-PEG-anti-HRP NPs). The NP-antibody conjugates were removed after 2 h and the cells were intensively washed. The images were taken 1 h later by employing a Zeiss fluorescent microscope. Images show the fluorescence and bright field channel, as the overlay of both channels.

For all formulations tested, more NPs were taken up if they were incubated with the cells in the absence of serum, which is consistent with previous findings [31]. We speculate that proteins and other constituents of serum that interact with the NPs change their properties in such a way that they are internalized by an endocytic pathway as it has been previously described [32]. Interestingly, the PEGylated NPs were taken up well by cells. This indicates that coating Au NPs with PEG does not completely preclude their internalization. In addition, NP-antibody conjugates were incorporated by cells, as shown in the fluorescence microscopyimages in Figure 12.

**Figure 13:**
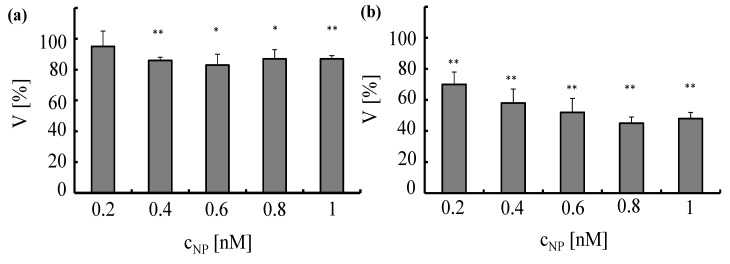
Toxicity induced by Au-PEG NPs in A549 cells (**a**) and HUVECs (**b**)Au-PEG NPs, at concentrations ranging from *c_NP_* = 0.2 to 1 nM, were incubated for 4 h with A549 cells or HUVECs in media containing serum. Cell viability (V) was determined 24 h after adding the NPs to the cells. The viability was normalized to 100% for untreated control cells, *p < 0.01; **p < 0.001(versus control value).

The toxic effect of the NPs to the cells was analyzed with a standard viability assay. Ten thousand cells per well were seeded in 96-well-plates one day before planned experiments. A549 cells and HUVECs were incubated for 4 h with Au-PEG NPs at different concentrations *c_NP_* ranging from 0.2 to 1 nM. Subsequently, the cells were intensively washed and further cultured. Cell viability was evaluated 24 h after NPs had been added to the cells by the MTT assay (Roche, Germany) according to the manufacturer’s instructions, *cf.* Figure 13. The assay is based on conversion of the tetrazolium dye 3-(4,5-dimethylthiazol-2-yl)-2.5-diphenyltetrazoliumbromide to its insoluble formazan, which is purple in color. Data demonstrate that Au-PEG NPs reduce cellular viability in a concentration-dependent manner.

### 2.7. Effect of Au NPs with Anti-VEGF on VEGF Stimulation of Cells

Under physiological conditions VEGF binds to its receptor (VEGFR) present on the membrane of HUVECs, which initiates cascades of signals that stimulate many processes including angiogenesis [33,34]. VEGF receptors convey information to other signal transduction molecules via autophosphorylation of distinct residues in their structure. If VEGF binds to its receptor, HUVECs proliferate. If one blocks the receptor with an antibody [33,35] or NP [36,37], there is reduced proliferation. Antibody-based therapies relay on a sequestering of VEGF molecules by specific antibodies. In this way, VEGF binding to its receptor is prevented [38].

**Figure 14:**
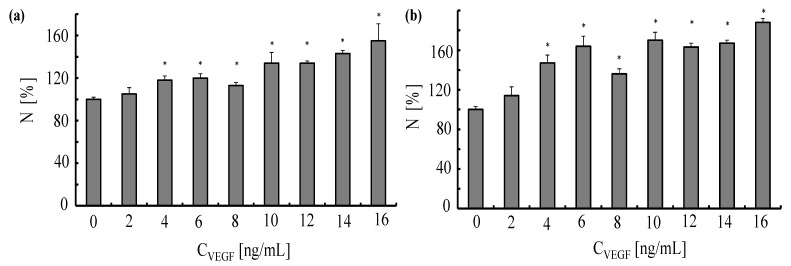
Response of human umbilical vein endothelial cells to vascular endothelial growth factor. The endothelial cells were plated in 96-well plates (5000 cells/well) one day before planned experiments. The cells were exposed for 24 h (**a**) and 48 h (**b**) to different concentrations *C*_VEGF_ of VEGF. The normalized numbers of cells *N* in culture were evaluated by performing a proliferation test. Data correspond to the mean value ± standard deviation obtained from *n* = 4 experiments, *p < 0.001 (versus control value, no VEGF).

In a first set of experiments, we tested the response of HUVECs to stimulation with VEGF. To that end, the cells were exposed for 24 or 48 h to VEGF at different concentrations (*C*_VEGF_ = 2–16 ng/mL). As demonstrated in Figure 14, VEGF stimulated proliferation of HUVECs in a dose-dependent manner. At concentrations ≥ 10 ng/mL the number of cells in culture increased by more than 20% after 24 h and by more than 50% after 48 h. Based on these results, we chose to stimulate HUVECs with VEGF at concentrations of 12 and 16 ng/mL in all subsequent experiments.

**Figure 15:**
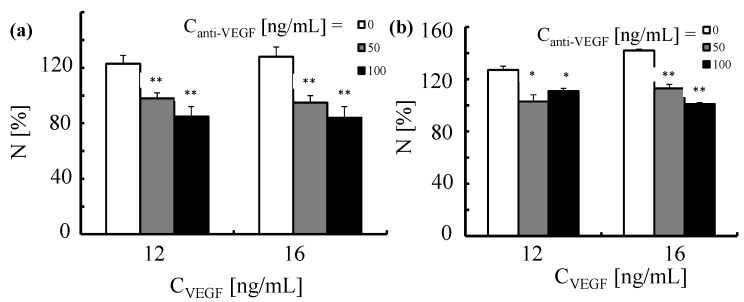
Proliferation of human umbilical vein endothelial cells triggered by VEGF and neutralization induced by anti-VEGF antibody. HUVECs were first exposed for 2 h to anti-VEGF antibody at two concentrations (*C*_anti-VEGF_ = 50 and 100 ng/mL). This was followed by the incubation with VEGF (*C*_VEGF_ = 12 and 16 ng/mL) for (**a**) 24 and (**b**) 48 h. The number of cells was normalized to 100% for untreated control cells. *p < 0.01; **p < 0.001(*versus* cells treated with VEGF only).

Next, in order to verify whether proliferation elicited by VEGF can be neutralized by anti-VEGF antibodies, we pre-incubated HUVECs with the antibody, which was followed by stimulation with VEGF. The results presented in Figure 15 demonstrate that soluble anti-VEGF antibodies inhibit proliferation of endothelial cells induced by VEGF in a dose dependent manner. Note that this is not due to blocking of the VEGF receptor but by binding of anti-VEGF to VEGF, which cancels the biological activity of VEGF.

We next assessed whether a similar effect could be achieved by the anti-VEGF antibodies attached to Au NPs (Au-PEG-anti-VEGF NPs). HUVECs were first incubated with Au-PEG-anti-VEGF NPs for 2 h. This was followed by the stimulation with VEGF for 24 and 48 h. To verify whether the observed effects were specific, in this set of experiments, we also tested Au NPs functionalized with the irrelevant antibody anti-HRP (Au-PEG-anti-HRP NPs).

**Figure 16:**
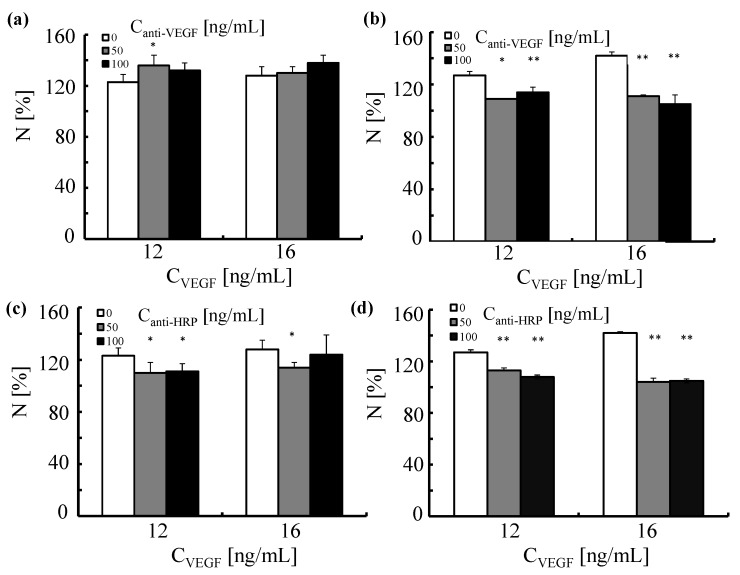
The effect of Au NPs functionalized with anti-VEGF or anti-HRP antibody on the proliferation of human umbilical vein endothelial cells triggered by VEGF. HUVECs were first exposed to Au NPs functionalized with (**a**,**b**) anti-VEGF or (**c**,**d**) anti-HRP antibodies. The NPs were added to the cells to reach concentrations of the antibodies equal to *C*_anti-VEGF_ and *C*_anti-HRP_ of 50 (grey bars) and 100 ng/L (black bars) (adjusted by the NP concentration by knowing the number R_P/NP_ of antibodies per NP as shown in Table 1). This was followed by the incubation with VEGF (*C*_VEGF_ = 12 and 16 ng/mL) for (**a**,**c**) 24h and (**b**,**d**) 48 h. The number of cells was normalized to 100% for untreated control cells. *p < 0.01; **p < 0.001 (*versus* cells treated with VEGF only).

As demonstrated in Figure 16, Au NPs functionalized with anti-VEGF antibody (Au-PEG-anti-VEGF NPs) exhibited some effect on the proliferation of HUVECs upon stimulation with VEGF over a longer period of time. However, the same trend was observed for NP carrying anti-HRP (Au-PEG-anti-HRP NPs). Therefore, it is likely that the recorded decrease in the number of cells in culture was not caused by a specific interaction of the functionalized Au NPs with VEGF but rather was associated with NP induced toxic effects on cells.

## 3. Conclusions

A protocol for functionalizing Au NPs with antibodies has been presented, together with characterization procedures, which quantify the number of antibodies per NP. It is demonstrated that biocojungation did not induce agglomeration. While successful bioconjugation could be demonstrated, this does not provide information about the biological activity of the attached antibodies. For this, profound characterization is also required. With the presented data, a biological effect of the NP-antibodies is demonstrated. However, this example demonstrates that such data can be misleading. As the same effect was observed with NP-antibody conjugates with an irrelevant antibody, the effect can’t be ascribed to a specific antibody effect but rather to general NP-induced toxicity. This example points out that antibodies can be deactivated, and that controls with irrelevant antibodies are required to demonstrate specific biological activity of NP-antibody conjugates.
